# The governance of traditional medicine and herbal remedies in the selected local markets of Western Kenya

**DOI:** 10.1186/s13002-020-00389-x

**Published:** 2020-06-26

**Authors:** Willy Kibet Chebii, John Kaunga Muthee, Karatu Kiemo

**Affiliations:** 1 Department of Plant Science and Crop Protection, Wangari Maathai Institute for Peace and Environmental Studies, P.0 Box 29053, Kangemi, Nairobi 00625 Kenya; 2 Department of Clinical Studies, Wangari Maathai Institute for Peace and Environmental Studies, P.O Box 30197, Nairobi, 00100 Kenya; 3 Department of Sociology, Wangari Maathai Institute for Peace and Environmental Studies, P.O Box 30197, Nairobi, 00100 Kenya

**Keywords:** Traditional medicine, Traditional governance practices, Modern governance practices, Market centres, Western Kenya

## Abstract

**Background:**

A lot of emphasis is often placed on modern governance systems and little or no attention is given to traditional governance practices which remain largely undocumented. The study aimed at finding out important traditional and modern governance practices that regulate traditional medicine sector in Western Kenya.

**Materials and methods:**

The study was carried out in selected market centres of Western Kenya where the identified traditional medicine practitioners (TMPs) sell their traditional medicine. All consenting TMPs and professional experts were interviewed with the aid of a semi-structured questionnaire. Purposive sampling design with elements of snowball techniques was employed in tracing competent traditional medicine (TM) experts and relevant professional experts. The data collected was processed in Microsoft Excel and descriptive statistics performed. Pearson’s chi-square statistics was carried out to determine the significance of the traditional and modern governance data sets using the STATA software.

**Results:**

Modern governance practices were not significantly different in all the market centres surveyed (*p* = 0.080). Equally, the traditional governance practices were also not significantly different in all the selected market centres (*p* = 1.000).

**Conclusions:**

Traditional governance practices play an important role in the governance of traditional medicine and are shaped by the socio-cultural beliefs of the local communities. Modern governance practices, on the other hand, are widely perceived as top downregulation of the traditional medicine growing industry.

## Introduction

Governance as used in the health systems refers to how decisions are made and implemented. It entails governance of healthcare services and policy. On a socio-ecological and environmental viewpoint, governance refers to means and ways on how collective decisions and goals are made and achieved. Key aspects of governance include transparency, participation, accountability, integrity and capacity [1, 2]. On the other hand, Traditional medicine (TM) is a sub-set of ethnomedicine and it entails the use of available resources (minerals, animal and plant materials) including medicinal plants useful in the treatment various diseases and ailments. This study focused on the broader context of TM as practised by the traditional medicine practitioners (TMPs) who are basically drug sellers and treat patients in the selected traditional medicine markets in Western Kenya [3–5]. The resurgence of interest in TM has been attributed to the challenges faced in the treatment of some chronic diseases and conditions using modern medicine [6–9]. The popularity of herbal medicines has also been attributed to the wider cultural belief that people are close to nature, their accessibility and affordability [10]. TM is also important for socio-economic, cultural and environmental benefits and in supporting livelihoods of the TMPs [11]. The renewed interest in TM draws impetus from increasing human population, changing lifestyles and standards of living among societies over time. In addition, various cultural practices promote the use of traditional medicine [4, 12]. Herbal medicines enjoy high acceptability among communities as they are considered cheap and of intense cultural attachment [10, 13].

Local market centres have become increasingly important in the trade and practice of traditional medicine. However, the magnitude of trade in traditional medicine and the existing governance systems are still not clear and remain largely undocumented [14]. Regular supply of traditional medicine can be achieved through sustainable practices ranging from conservation, cultivation, proper harvesting, regulated trade and controlled use. This requires a well-structured sustainable governance system [12]. On the governance of medicinal and genetic resources, the Nagoya Protocol of 2010 building on the Convention on Biological Diversity (CBD) advocates for the exchange and sharing of indigenous knowledge held by local communities and acknowledges the importance of fair and equitable sharing of available genetic resources. The Nagoya Protocol promotes sustainable development and conservation of biological diversity and in addition recognizes the importance of customary law and prior informed consent of members of local communities who are key sources of indigenous knowledge or traditional knowledge [15]. Traditional medicine has not been effectively mainstreamed in the overall Kenyan primary health care sector and still lack clear legal and policy guidelines [16].

Little is known about the traditional governance practices that help regulate the trade and practice of traditional medicine in Kenya. A lot of interest and focus has always been attached to modern governance practices ranging from constituted laws to policies that regulate traditional medicine. The governance of traditional medicine trade and practice in the devolved county market structures is not clearly stated and documented. This study attempted to find out the important traditional and modern governance practices that regulate the existing trade in traditional medicine and also assess the knowledge, attitudes and perceptions (KAP) of the TMPs drawn from the selected markets or trading locations in Western Kenya. Finally, there was a need to evaluate the significance of the traditional governance practices in traditional medicine trade and practice and assess whether they should be harmonized with modern governance practices.

### Governance of traditional medicine industry in Kenya

Generally speaking, governance expresses the organization of people and exercise of power whether formally or informally and the ability to formulate rules on how to attain set goals and objectives at local, social, institutional, national or global levels [17]. Traditional medicine practices can be governed to some extent through local customs and indigenous knowledge normally transferred via cultural means. However, there is a need to appreciate and harmonize the traditional systems of governance with the modern formulated policies, laws and by-laws passed by county and national governments. The push for integration of traditional medicine into the primary health care is necessitated by the inability of the modern health facilities in meeting the health demands of an increasing Kenyan population [18].

The demand for traditional medicine, particularly the medicinal plants, also opens avenues for conflict due to over-harvesting or bad harvesting practices [12]. Therefore, strong governance measures are invaluable in enabling controlled harvesting of medicinal plants including those sourced from the wild [19]. Functional local institutional policies play a vital role in the transfer of indigenous knowledge on traditional medicine [20]. It has been reported that traditional knowledge is important in the management of locally available natural resources [21, 22]. Nagoya Protocol advocates for fair and equitable sharing of these locally available natural and genetic resources with respect to indigenous knowledge, institutions and practices held by communities. The present laws and regulatory policies are thought to be less efficacious in the current trade and practice of traditional medicine and qualify the need for clear, robust, unambiguous and definitive legislation [23].

### Modern governance practices

Modern governance practices are guided and shaped by national laws, county by-laws, acts of parliament and policies. Three key items to consider information of good laws and policies include having the right definition of traditional medicine, robust regulations and the preservation of intellectual property rights [8]. The following acts of parliaments, laws, policies, bills and gazette regulations helped shaped the traditional medicine practice in Kenya as gathered from various sources (Appendix 1).

### Witchcraft act, 1925, cap 67 Laws of Kenya

The Witchcraft Act of 1925 outlawed any forms of witchcraft practices that were detrimental to the colonial government administration. Traditional medicine practitioners were generally labelled witchdoctors or often charged for practising witchcraft and being in possession of outlawed charms. The authentic traditional healers lived in constant fear and risked being convicted, punished or slapped with hefty fines or imprisonment. The Witchcraft Act therefore slowed down the advancement of traditional medicine in the pre-independent Kenya.

#### Alma Ata declaration, 1978

The Alma Ata international conference held in the former USSR advanced the agenda for primary health care for all and declared health a fundamental human right. It referred to gross inequality in health care as unacceptable and a cause of great concern. The declaration tasked governments with a responsibility to formulate policies, strategies and plans of action that promote the provision of primary health care. Alma Ata Declaration recognized and acknowledged the roles played by midwives, community health workers and traditional medicine practitioners in the provision of primary health care.

#### Development plan, 1989–1993

The Kenya’s Development Plan of 1989 recognized traditional medicine and set the agenda for the promotion of TMPs social welfare and work environment. The Ministry of Health and the Provincial Administration was tasked with the responsibility of ensuring that all practising TMPs have been registered.

#### Convention on biological diversity, United Nations, 1992

The 1992 Convention on Biological Diversity (CBD) advocated for the use of indigenous and traditional knowledge in the conservation of biodiversity, equitable sharing of benefits and sustainable use of natural resources. Annex I of the convention highlighted the importance of medicinal plants identification and more so the key indicator species that may be useful in research, conservation or consumption.

#### Kenya National Drug Policy, 1994

The Kenya National Drug Policy of 1994 acknowledged traditional medicine as a key component of Kenya’s culture and thus the need to mainstream it into the primary health care system.

#### Registration and recognition of TMPs, Ministry of Gender, sports, culture and social services, form DC1

The Ministry of Gender, Sports, Culture and Social services tasked the Department of Culture with the responsibility of vetting the Traditional Medicine Practitioners (Traditional Birth Attendants, Bone Setters, Traditional Surgeons, Herbalists and Medicinal Plant Conservationists) with assistance from local administration authorities. The Department of Culture spelt out the eligibility criteria which included submitting 3 to 6 drug samples, medicinal plant preparations or voucher plant specimens to recognized government and certified research institutions for laboratory analyses. The Department of Culture also outlined the registration guidelines for foreign groups or individuals dealing with traditional medicine

#### National policy on traditional medicine and regulation of herbal medicines, 2005

A World Health Organization (WHO) global survey report on Traditional Medicine/Complementary and Alternative Medicine (TM/CAM) of 2005 involving 141 member states of the overall 191 member states raised valuable concerns on the issue of safety, drug efficacy and quality control. Only few member states (32%) had developed a policy on TM/CAM and majority (61%) had established a registration system for herbal medicines. Kenya reported significant progress on the regulation of traditional medicine by setting up Kenya Medical Research Institute (KEMRI) in 1984 but missed out on the key policy requirements of having a national programme, national coordinating office, an expert committee, clear regulatory framework, national pharmacopoeia, national monograph, registration system and a solid manufacturing system.

#### Sessional paper on traditional medicine in Kenya (2009)

The Sessional Paper of 2009 on traditional medicine in Kenya anchored five key objectives that promoted traditional medicine namely, regulation, setting up of relevant institutions, contribution of traditional medicine in health care delivery, safety and efficacy and finally the ex situ and in situ conservation of medicinal plants. The paper also pointed out the information gap on the trade of medicinal plants, good manufacturing practices for herbal remedies/products and standardization procedures.

The Sessional Paper highlighted the enforcement of ethical principles in traditional medicine practice which includes equity, fairness and rights to access of medical care. It further recognized the contribution of communities and stakeholders in the use of medicinal plants and the critical aspect of benefit sharing. Finally, the paper proposed commercialization of traditional medicine, management of information disclosure and setting up of robust institutions, laws and policies to govern traditional medicine in Kenya.

#### Registration of herbal and complementary products

##### Pharmacy and poisons board (2010)

The Pharmacy and Poisons Board (PPB) document provided guidelines for submission of traditional herbal and complementary products for registration and licensing. Eligible applicants are required to present; 3 drug samples, a Certificate of Analysis from an accredited research institution, a Certificate of the Pharmaceutical Product and a brief descriptions of the dosage forms (i.e. macerate, infusion, ash and solutions), plant part utilized, means of harvesting/collection, drying, storage and preservation methods, efficacy of the product over time and lastly the applicant declaration.

##### The traditional medicine and medicinal plants bill, 2010

The Traditional Medicine and Medicinal Plants Bill of 2010 laid out proper definitions for traditional medicine and medicinal plants. Traditional medicine was defined as a finished and labelled medicinal product that contains an active ingredient, aerial or underground plant parts in crude or processed form. On the other hand, a medicinal plant was defined as a plant that contains a therapeutic substance or a plant that serves as a precursor for the synthesis of useful drugs. The bill proposed the creation of a Traditional Medicine Management Council (TMMC) that was to oversee the practice of traditional medicine in Kenya. TMMC was to draw representation from the Ministry of Agriculture, National Environment Management Authority (NEMA), Kenya Bureau of Standards (KEBS), Kenya Plant Health Inspectorate Services (KEPHIS), Kenya Medical Research Institute (KEMRI), National Council for Science and Technology (NCST) and Kenya Industrial Property Institute (KIPI).

The bill underscored the importance of domestication of wild medicinal plants, protection of intellectual property rights (IPR) and Indigenous Knowledge (IK). It also set out the eligibility criteria for recognition and certification of traditional medicine practitioners. Eligible candidates are required to have acquired formal knowledge in traditional medicine or have completed relevant training. This requirement is a tall order for most TMPs and was considered elitist by many traditional practitioners who are largely informal and have acquired basic education or no formal education at all. The bill further proposed a penalty or punishment for rogue traditional medicine practitioners.

##### The health bill, 2012

The Health Bill of 2012 recognized the role of traditional and complementary medicines in the health care sector. It defined a health care professional as an individual with professional training or adequate qualifications for provision of medical services. It also defined traditional medicine as products extracted from plants, animals or mineral sources, prepared and administered based on traditional knowledge.

The bill also proposed the formation of a Kenya Health Services Authority (KHSA) of which one traditional and complementary expert was to be appointed as a member of the authority. The bill also empowered the Cabinet Secretary of Health and in consultations with the proposed KHSA provide regulations for better governance of traditional medicines.

##### The traditional health practitioners bill, 2014

The Traditional Health Practitioners Bill of 2014 provided provisions for training, registration and licensing of the traditional health practitioners. It defined traditional health practice (THP) as the utilization of traditional medicine with the aim of diagnosis, treatment or prevention of an illness. It also proposed the establishment of a Traditional Health Practitioner Council of Kenya (THPCK) of which three experienced traditional health practitioners with over 5 years of practice were to serve in the council. The bill further provided eligibility criteria for practitioners and applicants were expected to have accomplished a training employment for over one year under supervision from a competent traditional health practitioner.

##### The health bill, 2015

The Health Bill of 2015 explicitly expressed the richness of traditional medicine in terms of transfer of knowledge, skills and practices in the provision of healthcare. Furthermore, it expressed optimism in the ability of traditional medicine regarding prevention, diagnosis and treatment of diseases. It also expressed the need for sound policies that may help regulate the practice of traditional medicine through the Department of Health or national government. The bill engendered a patient referral system where traditional healers could refer patients to modern healthcare facilities. Finally, the bill proposed the creation of a National Research for Health Committee (NRHC) of which one traditional medicine expert was to be a member.

##### Protection of traditional knowledge and cultural expressions act, no. 33 of 2016

The act made provisions for the protection of traditional medicine knowledge, genetic resources and biological diversity. Most of the TK is passed orally passed from one generation to another and can easily be lost or distorted.

##### The health act no. 21 of 2017

The Health Act No. 21 of 2017 empowered the Department of Health to provide policies and regulatory institutions that guide the practice of traditional and alternative medicine. The regulatory bodies created shall provide guidance on registration, licensing and standards compliance. The act further provided mechanisms for Traditional Health Practitioners to refer patients to modern health care facilities.

##### The health Laws (amendment) bill, 2018 Kenya gazette supplement no. 36, National Assembly bills, no. 14.

The Health Laws amendment bill of 2018 recognized traditional and alternative medicine as a health product.

##### Traditional and alternative medicine policy draft, 2018, Ministry of Health

The Traditional and Alternative Medicine Policy draft proposed provisions for mainstreaming Traditional and Alternative Medicine into the National Health Care System to boost access to health care for all. The policy draft highlighted strategies that underscore the need and importance of biodiversity conservation, sustainable harvesting and cultivation; safety, efficacy and quality; education and training; proper use and quality assurance; standardization of traditional medicine; good manufacturing practices; ethical principles; equity; protection of intellectual property rights; access and benefit sharing; commercialization of TM; and, lastly, issues of disclosure and secrecy. The policy draft encouraged documentation and recording of traditional medicine knowledge and setting up of digital traditional medicine libraries.

The policy draft made provisions for the setting up of legal and institutional frameworks of traditional and alternative medicine, and National Traditional and Alternative Practitioners Council (NTAPC) tasked with the responsibility of registration, regulation and development of standards.

##### The traditional and alternative health practitioners bill, 2019

The bill provided for training, registration and licensing of traditional and alternative health practitioners and spelt out regulatory and disciplinary guidelines. The bill made provisions for the development of the Traditional and Alternative Health Practitioners Council (TAHPC) of which two registered traditional health practitioners with over 10 years’ experience would be selected as members of the council.

##### The health Laws (amendment) act, no. 5 of 2019

The recently passed Health Laws Act No. 5 of 2019 recognized traditional medicine as a health product.

### Traditional governance practices

The space for traditional health practitioners in Kenya is provided for by the increasing demand for medicinal plant products. The cultures and traditions of various communities and societies shape the utilization of these medicinal plant materials [3]. In India, traditional health care systems are relevant and aid in the treatment of chronic illnesses. Traditional health care systems provide space for institutional networking, bio-prospecting and in fighting biopiracy. Recognized local health care traditional practitioners include traditional birth attendants, bone setters, and experts of snakebite treatment among others. Family traditions and culture influences the choice and selection of an appropriate health care system [8]. Practices of traditional and complementary systems of medicine are deeply rooted in the cultural environment, community beliefs, emotions, life experiences, spiritual considerations and even religion [24, 25]. Traditional medicine governance practices therefore consist of culturally binding customs, taboos, beliefs and societal informal regulations that silently regulate TM and are informally passed over generations. Going against the cultural norms, customs and taboos presumably attract punishment from the gods and the spiritual world. TMPs strictly observe these informal traditional governance regulations to successfully practice in traditional medicine.

### Standardization of traditional medicine

Standardization of traditional medicine refers to the development and application of standards to critical elements of traditional medicine that include medical care, research, industry and culture in order to ensure the maintenance of quality, safety, and modernization. Standardization is measured using the quality of raw materials, process controls, manufacturing process and validation. The quality of raw materials is influenced by geographical origin, plant parts used, collection period and hygiene conditions. China has made significant progress in terms of setting up proper standardization measures for its traditional medicine popularly dubbed traditional chinese medicine (TCM) which has over the years gained global prominence. Rapid advancement in Chinese TCM standardization is enabled through direct state support via the State Council of China and the Ministry of Science and Technology in ensuring safety and efficacy [6, 26, 27]. The process towards full legalization, legitimization and professionalization of traditional medicine requires scientific research, testing and validation with effective oversight from robust government authorities. Government oversight entails accreditation, licensing and education programs for traditional medicine practitioners [28].

### Challenges faced in traditional medicine

The growing traditional medicine industry associated with limited knowledge on medicinal properties comes with a myriad of safety and health concerns. China and Japan are on the forefront when it comes to the integration of traditional medicine or herbal remedies into the primary health care system. However, in the African context, traditional medicine practitioners do not disclose vital information about their trade to patients or even researchers. Most TMPs are ignorant of the possibility of herbal interactions that may alter drug efficacy or cause adverse reactions. Herbal-conventional drug interactions may disrupt drug absorption and metabolism [29, 30]. A case study in Ghana (Kumasi South Hospital) revealed that most biomedical practitioners are skeptical about the integration of traditional medicine. Positive integration of traditional medicine requires robust regulatory policies and protocols for integration [31] (Table 1).

**Table 1:** Problems and issues associated with the use of herbal medicines. Drawn from [7, 30]

Problem/challenge	Issues to be sorted out
1. Quality and purity	Adulteration, plants misidentification, drug preparations and formulations
2. Processing and harvesting	Poor harvesting practices and processing techniques, contamination
3. Quality control	Standardization, poor manufacturing practices
4. Administrative issues	Regulation and control, proper monitoring efforts
5. Infrastructure	Processing techniques, trained personnel, product approval, post-market surveillance
6. Pharmacovigilance	Adverse reactions, contraindications, drug interactions
7. Clinical trials	Safety and efficacy
8. IPR and biopiracy	Proper documentation of TK and folk knowledge
9. Research and development	Mode of action of drugs
10. Others	Unethical practices, quacks (incompetent TMPs), inadequate funds, poor marketing, knowledge sharing, biodiversity protection, conservation and protection of medicinal plants

Lack of cooperation and collaboration between traditional healers and biomedical practitioners is a huge impediment towards integration of traditional medicine. Consumers choose traditional medicine because they identify and share common traditional culture, beliefs, relationships, social life and environment with the traditional medicine practitioners. The traditional medicine users’ belief traditional medicine healers are more approachable, accessible and their drugs affordable as compared to modern medicine [32]. Cooperation between traditional and allopathic practitioners is touted to be beneficial and complementary to health care delivery but often derailed by suspicion [33]. On drug management, toxicity (hepatoxicity and cardiotoxicity) cases in traditional medicine use have been reported though not widely documented [6, 25, 34]. In addition, it is hard to quantify the actual trade in medicinal plant products in the markets based on complexity and informal nature of traditional medicine markets. It is also hard to project the economics involved in such a subsistence-based trade largely conducted in open-air markets [3].

In most countries of sub-Sahara Africa, the problem of decreasing agricultural and rural land sizes, heightened extraction and poor harvesting practices has had a negative effect on traditional medicine supplies [3, 35].

### Formal and informal nature of traditional medicine markets

Efforts to formalize traditional medicine which is largely an informal industry has gathered momentum and prominence in the developing countries. For instance, a certified traditional medicine trader in developing countries seeking markets in Europe is required to submit a formal dossier showing a scientific proof and empirical evidence of the herbal medicine’s safety and efficacy. The regulations in the USA are even stricter since theoretical claims for herbal remedies are not permitted. For developing nations, relaxed regulatory procedures and legal lacunae have created an environment where unregistered products can easily find their way into local markets. WHO cites rational use of traditional medicine as a major global policy challenge [36–39]. In Indonesia, the traditional herbal medicine (*Jamu*) is informally sold on the streets and markets of Java city. There is no sufficient data to categorically state the active constituents of the commonly used traditional medicine [40]. Pakistanis have made huge strides in the use of traditional medicine by promoting and recognizing formal complementary and alternative medicine (*Unani*) teaching institutions [41]. In most parts of Africa, it has been increasingly difficult to quantify the volume of trade-in medicinal and aromatic plants (MAPs) in what has been termed an informal or hidden economy [42].

Documentation of the activity and efficacy of medicinal plants is a viable route towards formalization as opposed to the informal oral traditions. These oral traditions are prone to loss or distortion as the original traditional medical knowledge is passed from one generation to the next [43]. The acceptance of the health benefits of traditional medicine and subsequent integration into the formal healthcare system has been globally detracted by widespread negative perceptions attached to traditional medicine [44]. Ecological factors and sustainability goals have further pushed the formalization of natural products trade agenda. These factors include conservation of wild medicinal plant species, local leadership, benefits sharing and desire by local authorities to generate revenue. Alienated pathways to formalization of trade legislations and policies excluding major stakeholders have led to unintended negative consequences as a result of over-regulation and poorly formatted laws. Unintended consequences of elite formalization include marginalization of small traders, elite capture, exploitation of trade-in political boundaries, costly levies, burdening bureaucratic processes, heightened conflicts among major players, ballooning corruption and sexual exploitation of women traders [45].

Lastly, the absence of formal processes of traditional medicine may escalate safety concerns, aid trade malpractices and facilitate loss of traditional medicine knowledge. Formalization of traditional medicine and linking it to formal health care systems promote determination of medical effects of medicinal plants in terms of diseases and their symptoms. Formal environment promote sharing of medical knowledge, drug discoveries and better understanding of pathology and ethno-pharmacology [46].

### Secrecy and suspicion

Secrecy of the traditional medicine knowledge or practice poses a major challenge in the advancement of traditional knowledge and research [11, 35]. Some TMPs violate this secrecy and disclose some information in exchange of monetary gain or as a source of livelihood [47]. **S**ecrecy is often fuelled by mistrust between major traditional medicine stakeholders and driven by fear of losing inherited traditional medicine knowledge [48].

## Methods

### Study area

The study was conducted in the purposively selected markets found in the Western part of Kenya namely Kitale and Moi’s Bridge (Trans Nzoia County), Makutano (West Pokot County), Eldoret (Uasin Gishu County), Arror (Elgeyo Marakwet County), Luanda (Vihiga), Yala (Siaya County) and Kakamega (Kakamega County). The selection of these local markets followed guidance from professional experts in key government ministries or departments (Ministry of Health and the Department of Culture) who work closely with TMPs. The sites chosen fulfilled two important criteria: first, the presence of a competent traditional medicine practitioner and secondly, a functional traditional medicine market. The lead traditional medicine practitioners in the selected local markets recruited other practitioners and traditional medicine traders into the survey via a competent snowball process. Leading professional experts stationed in the Kenya’s capital city having direct involvement in traditional medicine were also interviewed. The selected traditional medicine markets reflected various tribal cultures and traditional medicine of the people of Western Kenya.

**Fig. 1:**
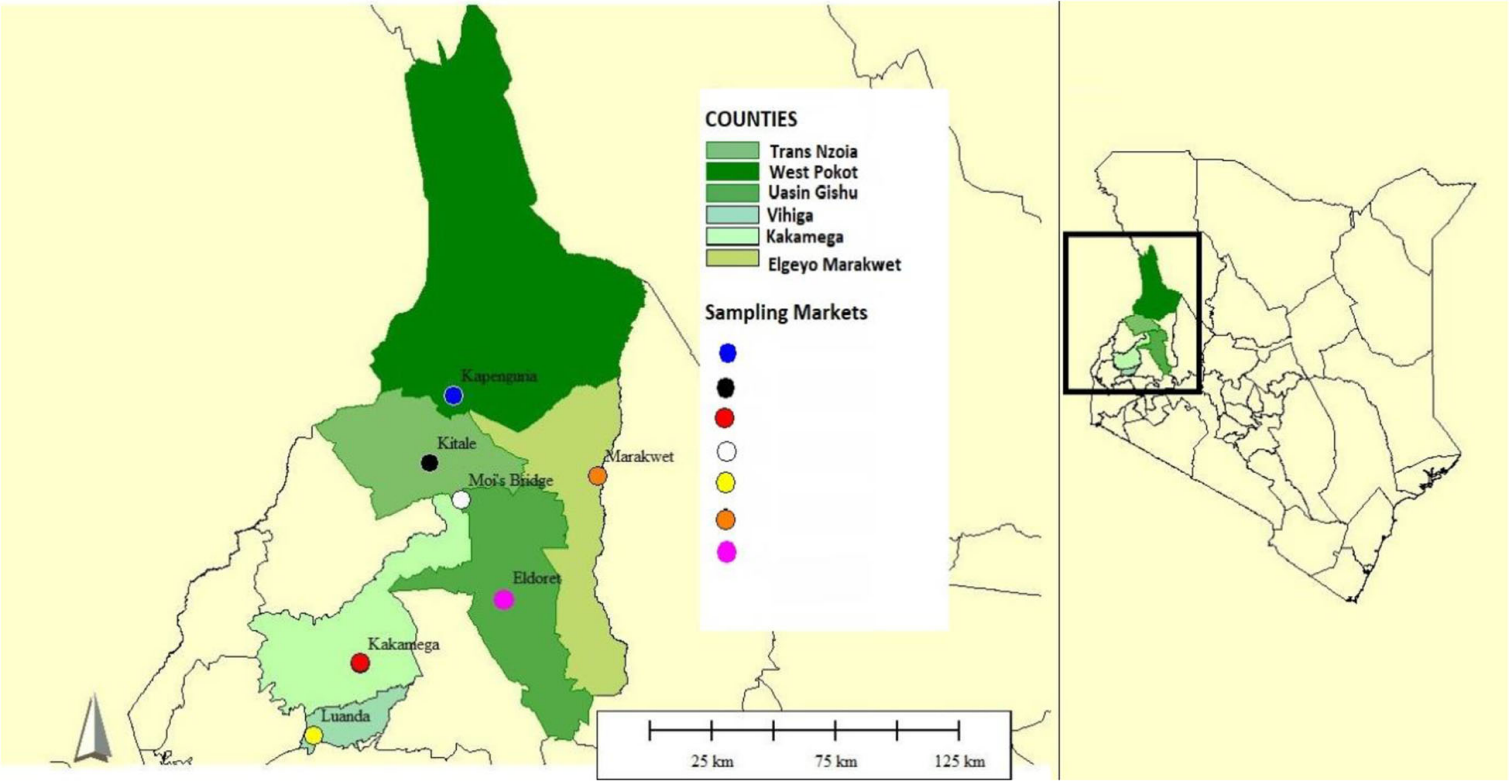
A map showing selected counties of Western Kenya and the sampled market locations

The population and demographic statistics of the counties containing the eight (8) selected traditional medicine markets (KNBS 2019) are tabulated in Table 2. The sampling map (Fig. 1) was generated in QGis 3.6.4 by importing field acquired GPS location coordinates using Garmin *etre* 20X version (Appendix 2) where oral/semi-structured interviews were undertaken upon attaining oral prior informed consents.

**Table 2:** Sampled counties showing the human population figures, population density and sex statistics including the intersex persons **(**KNBS 2019)

No.	Administrative County	Total human population	No. of males	No. of females	No. of intersex persons	Land area (km^**2**^)	No. of households	Density Persons/km^**2**^
1	Kakamega	1,867,579	897,133	970,406	40	3017	433,207	619
2	Uasin Gishu	1,163,186	580,269	582,889	28	3399	304,943	342
3	Siaya	993,183	471,669	521,496	18	2530	250,698	393
4	Trans Nzoia	990,341	489,107	501,206	28	2495	223,808	397
5	West Pokot	621,241	307,013	314,213	15	9123	116,182	68
6	Vihiga	590,013	283,678	306,323	12	564	143,365	1047
7	Elgeyo Marakwet	454,480	227,317	227,151	12	3032	99,861	150
	**Grand Totals**	**6,680,023**	**3,256,186**	**3,423,684**	**153**

The Kitale market is found in the Trans Nzoia County and serves as the main administrative centre of the county. Trans Nzoia County is predominantly an agricultural zone dominated by ferrasol soil type. It exhibits a unimodal rainfall pattern receiving about 1000–1200 mm per year and experiences a highland equatorial climate. The county has an altitudinal elevation of about 1800 m asl. After attaining independence from Britain in 1964, Trans Nzoia witnessed an influx of people from diverse Kenyan cultures who then settled in the former White Highlands as new farmers or farm workers. Maize and dairy farming contributes largely to the economy of Trans Nzoia County and the area has long been referred as the ‘food basket’ of Kenya. Tellegen and Foeken referred to the area as the ‘Maize granary’ of Kenya. Other crops cultivated in Trans Nzoia County include wheat, sunflower, tea, coffee and oranges. The area is dominated by the Luhya tribe (mostly from the Bukusu sub-tribe) followed by the Kalenjin and the Kikuyu tribes respectively [49–52].

The Eldoret market is found in the Uasin Gishu County and is the main administrative centre of the county. Uasin Gishu County measures about 3327.8 km^2^ with an elevation of about 1800 m asl. Rainfall amounts received ranges from 900 to 1200 mm per year [53–56]. The county is well known for its rich agriculture potential, particularly for milk and wheat production [57]. The economy of Uasin Gishu County also depends on maize, pyrethrum and her textile industry. Nitisols, acrisols and ferrasols are the dominant soil types of the county with few areas recording gleysols. The county was a White Highlands in the pre-independent Kenya and after independence, it also became a home to diverse tribal cultures. The Kalenjin sub-tribes of Nandi and Keiyo are dominant in Uasin Gishu. Kalenjins are known to produce the world’s fine athletes and long-distance runners [54, 56, 58, 59].

**Fig. 2:**
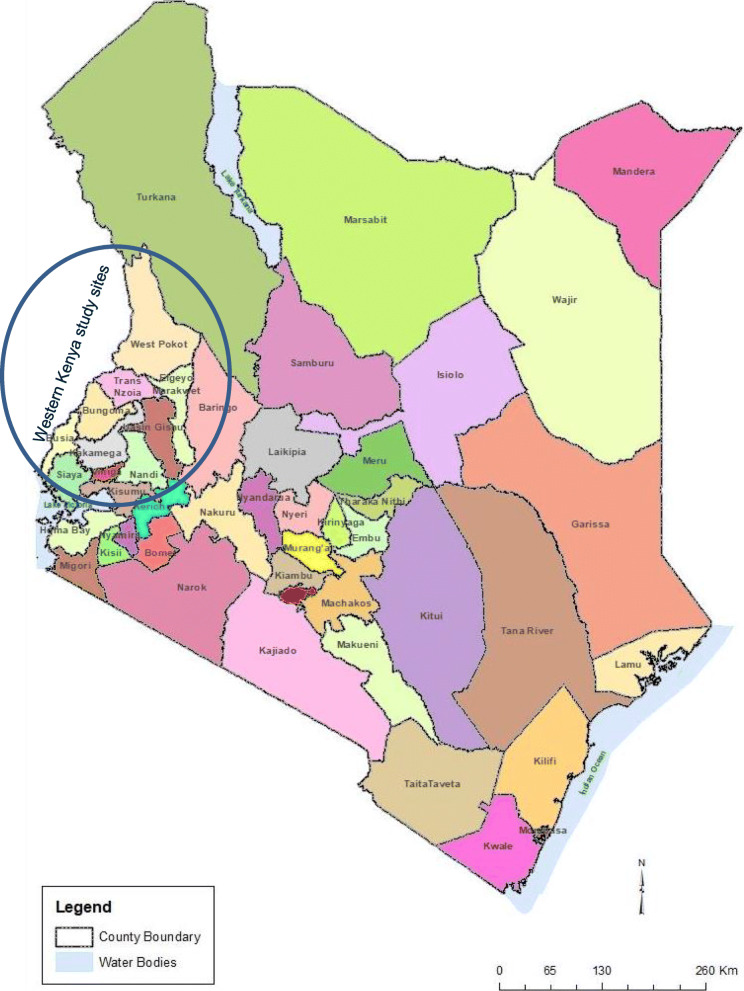
A map of Kenya adapted from KNBS 2019 https://www.knbs.or.ke/ showing surveyed medicine markets of Western Kenya

The Moi’s Bridge Market is a transboundary market found between the Trans Nzoia County and the Uasin Gishu County (Fig. 2).

The Arror market is found in the Elgeyo Marakwet County. The county is dominated by the two major Kalenjin sub-tribes of Keiyo and Marakwet. Mixed farming is commonly practised in the county with maize and dairy farming ranking higher. The former Marakwet District has a high number of authentic TMPs who practice within and without the county and some serving as suppliers of traditional medicine to other parts of the country [59].

The Makutano Market is found in the West Pokot County which borders the western Kenya counties of Elgeyo Marakwet, Trans Nzoia and the Republic of Uganda. It experiences two rainfall seasons in a year registering about 700–1600 mm per annum. The county geographically varies from arid to semi-arid climate with temperatures in the lowlands soaring up to 30 °C whereas the highlands getting as low as 9 °C. The Pokot sub-tribe of the Kalenjin tribe dominate the county [60, 61].

The Kakamega market is located in the Kakamega County and is the main administrative centre of the county. The county borders Trans Nzoia County to the North East, Uasin Gishu County to the East, Vihiga County to the South and Siaya County to the South West. The county has a dominant Luhya tribe population and home to the iconic Kakamega Forest (0° 19′ N, 34° 52′ E; altitude 1580 m asl) which is a Guineo-Congolian rain forest in Western Kenya and among the few Afromontane biodiversity hotspots of the world rich in flora and fauna [62–64]. From all the selected market sites, Kakamega has the highest human population (Table 2) with rich agricultural soils. However, most households have small farm sizes (an average of 0.7 ha/household) characterized by intensive farming of maize, sugarcane, vegetables and livestock. The precipitation levels received exceed 1500 mm per year [63].

The Luanda market is found in Vihiga County which has a dominant Luhya population. Geographically, Vihiga County borders Kakamega County to the North and Siaya County to the South West [65]. It has an elevation of 1750–2000 m asl and experiences a bimodal pattern of rainfall in the range of 1800–2200 mm per year. Despite the high rainfall amounts received, the area has low fertile soils mainly nitisols, ferralsols and acrisols with low water handling capacity. Vihiga County has the highest population density among all the selected study areas and most households have smaller farm sizes (an average of 0.6 ha) characterized by mixed crop/livestock farming [66]. Due to this evident population pressure, the area is prone to soil degradation and food insecurity [67, 68].

Lastly, the Yala market is found in the Siaya County which is positioned North of Lake Victoria and it is predominantly inhabited by members of the Luo Community. It has an elevation of about 1400 m asl and experiences an annual rainfall in the range of 800–2000 mm per annum. The soils of Siaya are well-drained and friable to shallow over petro ferric layers and are mainly nitisols, orthic ferralsols and acrisols [69, 70].

The Kenyan Census statistics of 2019 (Table 2) shows that Vihiga County is the smallest of the surveyed counties and measure about 564 km^2^ with the highest population density of 1047 persons per km^2^. West Pokot County is the largest county in land mass among the surveyed counties and measures about 9123 km^2^. Based on the total human population, Kakamega County leads with 1,867,579 people and least populated county being the Elgeyo Marakwet County with 454,480 people [KNBS 2019].

### Research design

The study used a purposive sampling with elements of snowball in the selection of the key respondents who then recruited other competent respondents into the survey [3, 33, 71]. All willing respondents were interviewed after obtaining an oral prior informed consent. Purposive sampling takes care of situations where some members of the target population may not be willing to participate in the subject, in this case, the fear of losing their unpatented traditional knowledge and medicinal products [72]. A flexible semi-structured questionnaire was used to gather data from the willing respondents in all the selected market centres in the Western Kenya region and relevant professional experts in Nairobi [32, 73, 74].

### Sampling frame and target population

Twelve (12) professional experts from Nairobi drawn from various ministries and government departments including twenty-seven (27) traditional medicine practitioners were interviewed (Table 3). This sampling frame was designed to capture both the professional (formal knowledge) and traditional medicine (informal knowledge) and existing governance practices (modern and traditional) evident in the traditional medicine markets of Western Kenya (Tables 3 and 4).

**Table 3:** Professional experts and traditional expert respondents drawn from a diverse array of specializations relevant to traditional medicine

Professional expert/traditional expert	Institution/ministry	Number interviewed
1. Pharmacist	Ministry of Health (MoH)	1
2. Cultural officer	Ministry of Culture, Sports and Arts (MoCSA)	1
3. Conservation expert	Kenya Forest Service (KFS)	1
4. Plant Taxonomist & Botanist	University of Nairobi	1
5. Pharmacognosy expert	University of Nairobi	1
6. Medical physiologist	University of Nairobi	1
7. Scientist	Kenya Forest Institute (KEFRI)	1
8. Scientist	Kenya Medical Research Institute (KEMRI)	1
9. Phytochemist	Kenya Forestry Institute (KEFRI)	1
10. Branding specialist	Kenya Industrial Property Institute (KIPI)	1
11. Environmentalist	National Environment Management Authority of Kenya (NEMA)	1
12. Quality Assurance Officer	Kenya Bureau of Standards (KEBS)	1
13. Traditional Medicine Practitioners	Ministry of Culture, Sports and Arts	27

**Table 4:** Selected market centres, willing respondents, their sex and ethnic affiliation of the TMPs. The number of respondents who refused to be interviewed was also captured

Market centre (county)	Number of Interviewed respondents (sex)	Ethnic affiliation of the traditional medicine practitioner/traders/ sellers (frequency)	Number of practitioners who refused to be interviewed	Ethnic representation of the buyers	Language(s) used in the informal markets
Eldoret (Uasin Gishu)	3 (all females)	Kalenjin (3)	1	Mostly Kalenjin	Kalenjin (Marakwet, Keiyo, Nandi dialects) and Swahili
Kitale (Trans Nzoia)	5 (all males)	Luhya (4), Swahili (1)	0	All tribes	Luhya (Bukusu) and Swahili
Makutano (West Pokot)	3 (all females)	Kalenjin (3)	6	Mostly Pokot	Kalenjin (Pokot) and Swahili
Moi’s Bridge (Trans Nzoia)	1 (male)	Maasai (1)	0	All tribes	Swahili, species referred to in Maasai
Arror (Elgeyo Marakwet)	2 (1 male, 1 female)	Kalenjin (2)	0	Mostly Kalenjin	Kalenjin (Marakwet) and Swahili
Kakamega (Kakamega)	5 (1 female, 4 males)	Luhya (5)	2	Mostly Luhya	Luhya and Swahili
Luanda (Vihiga)	6 (1 male, 5 females)	Luhya (6)	13	Mostly Luhya	Luhya and Swahili
Yala (Siaya)	1 (female)	Luo (1)	0	Mostly Luo	Luo, Luhya and Swahili

The choice of interviewing both traditional medicine experts and professional experts was to have a holistic picture of the trade-in traditional medicine in Kenya, particularly in the Western Kenya region. Most of the TMPs had little formal education, rich in traditional medicine knowledge and dominated by the older traditional medicine healers. On the other hand, professional experts were more formal, more educated, registered members of professional associations and are knowledgeable in allopathic medicine.

### Data collection

Data was collected through a mixture of methods which included field observations, photographs, field visits and interviews using a semi-structured questionnaire after obtaining an oral prior informed consent. The survey data was collected from February 2019 to September 2019 [4, 12, 35, 75–79]. Open-ended questionnaire was also used to enable the gathering of more information on the study thematic categories [35, 80].

Swahili is a national language and it is understood by almost every Kenyan citizen and was used in all the informal interviews with all traditional medicine practitioner and later translated into English. The English language was only used in formal interviews with professional experts. Due to low literacy levels of most of the traditional medicine traders, business transactions in the markets are conducted mostly using local/vernacular languages of the dominant tribes of those medicine markets. Almost all the traditional medicine sold in the markets have local/vernacular names except for species which are not indigenous.

### Cultural uniqueness and traditional medicine trade in Western Kenya

Traditional governance practices are perceived to be a product of socio-cultural norms and beliefs of the local communities and diverse cultures of the people. The traditional medicine practitioners of a particular area are unique and tend to have a specific traditional medicine or their combinations for treating specific diseases. TMPs ailing from one area tend to be governed or regulated by similar traditional rules and customs based on their respective cultures. Culturally speaking, the Luo traditions acknowledges social causation of disease ailments and link many diseases to sorcery and roaming spirits. The Luo TMPs more often than not employ a cocktail of magic, ritual and herbalism. Most Luo women TMPs serve as shrewd herbalists whereas the Luo men TMPs lead in magic and ritual activities. Traditional Luo medicine and practice presents a perfect blend of psychosocial and physiological components [69]. Communally shared traditional medicine knowledge of the Luo people of Siaya County enabled ordinary women treat common illnesses affecting household members (mostly children) whereas the professionally competent traditional healers are consulted for more serious, special or secret treatments [81].

Luhya folklore, particularly of the Bukusu, believes diseases are caused by environmental changes, certain surreal powers, curses, spells, evil eye, bad omen and evil spirits. Healing is sought from recognized traditional medicine experts who mostly use herbal remedies, rituals and/or traditional prayers [82]. For the Luhya communities of Kakamega and its environs, family institutions were credited for the transfer and preservation of traditional medicine knowledge and over time female practitioners have dominated the practice. Women of child-bearing age were barred from practising traditional medicine and were warned not to break the traditional regulations to avoid curse or infertility. Gender roles are clearly defined and evidently most women seek treatment from female practitioners for certain conditions, for instance, menstrual pain and men seek treatment from male practitioners over male-related dysfunctions [83]. Luhya is the second populous community in Kenya (Table 2) and it is a conglomeration of many sub-tribes namely Banyore, Batsotso, Abawanga, Tiriki, Maragoli, Samia, Bukusu, Tachoni, Idakho, Isukha, Kisa, Marachi, Marama, Kabras and Abakhayo and are dominant in the Kakamega, Vihiga, Busia, Bungoma and Trans Nzoia counties.

The Kalenjin tribe form the third most populous community in Kenya after the Kikuyu and Luhya (Table 2) and is made up of many sub-tribes namely Kipsigis, Nandi, Keiyo, Tugen, Marakwet, Pokot, Sabaot/Bongomek/Kony, Sengwer, Cherang’any, Terik and other tribal sub-divisions like Endorois, Samor, Lembus and Arror. The Kalenjins are dominant in the Rift Valley counties of Uasin Gishu, Kericho, Bomet, Nandi, Elgeyo Marakwet and the West Pokot (KNBS 2019). The Pokot Kalenjins, just like many other Kenyan tribal cultures, attributed diseases to spiritual, physiological, traditional curses and environmental causes [84].

Gender specializations have become so evident in many cultural groupings; majority of Marakwet women TMPs have specialized on diarrhoea, children diseases and abortion whereas male TMPs specialized in hypertension, rheumatism, craniotomy, tonsillectomy and bone setting [22]. From the 2019 Kenyan census statistics, the Luo comprised about 5,066,966 people was the fourth most populous tribe in Kenya whereas the culturally rich Maasai numbered 1,189,522. The Mijikenda tribe of the Kenyan coast mainly use the sacred Kaya forests to conduct their cleansing rituals and ceremonies. Some of the coastal medicinal plant species have been used for magical purposes, and others are psycho-medicines [85].

The Maasai are among the leading cultural communities of Kenya and Tanzania and are globally recognized for preservation of culture and traditional lifestyles. They are dominant in the Kajiado and Narok counties of Kenya and are pastoralists. Due to movements of TMPs across regions, a TMP from the coastal culture could practice in another county and earn livelihood.

### Data analysis

The data collected was entered in Microsoft Excel where descriptive statistics, mean, frequencies and percentage descriptive statistics, were performed [35]. The chi-square statistics was performed on the traditional and modern governance data sets using the STATA software version 13.0. The data presentation was done using tables, bar charts, column graphs and a pie chart [3, 86].

## Results

### Socio-economic and demographic characteristics of the traditional nedicine practitioners

Most of the traditional medicine practitioners (73%) were not aware of the existing laws and policies that regulate the traditional medicine but were fully aware of the county regulations, for instance, the mandatory trading fee and punishment for defaulters. Eighty-five percent (85%) of the sampled traditional medicine practitioners did not provide evidence of the Certificate of Recognition/Registration but fully complied with the county by-laws. The average daily trading fee for all the selected market centres was KES, 30 (Table 5).

**Table 5:** Socio-economic and demographic characteristics of the TMPs in the selected medicine markets of Western Kenya.

Variables	Survey response: percentage (%), number (*N*) and means
1. Number of TMPs interviewed	27
2. Mean age of TMPs	64 years
3. Average years of practice of TMPs (experience)	25 years
4. Percentage of willing interviewees	54%
5. Sex:	
Males	46%
Females	54%
6. Main category of traditional medicine clients	Reproductive age
7. Mean practising fee charged per day in KES.	KES. 30.00
8. Percentage awareness of existing laws and policies on TM	27%
9. TMPs recognized/registered by the Department of Culture	15%
10. Average monthly earnings	KES. 14, 269.00
11. TMPs with extra income-generating activities: selling calabash, bead, sweets, cigarettes, honey, tobacco powder for sniffing, imported and packaged herbal products, crop and livestock farming	35%

The mean age of the traditional medicine practitioners was 64 years with the average years of practice being 25, where the oldest practitioner was 85 years old with 48 years of experience. The youngest traditional medical practitioner was 30 years old with only 5 years of experience. Most of the traditional medicine practitioners (65%) had no additional income sources and solely depended on traditional medicine trade for livelihood. A considerable percentage of TMPs (47%) are not willing to be interviewed or share information.

### Modern governance practices

Pearson's chi-square (48) = 62.3438, *p* = 0.080

**Fig. 3:**
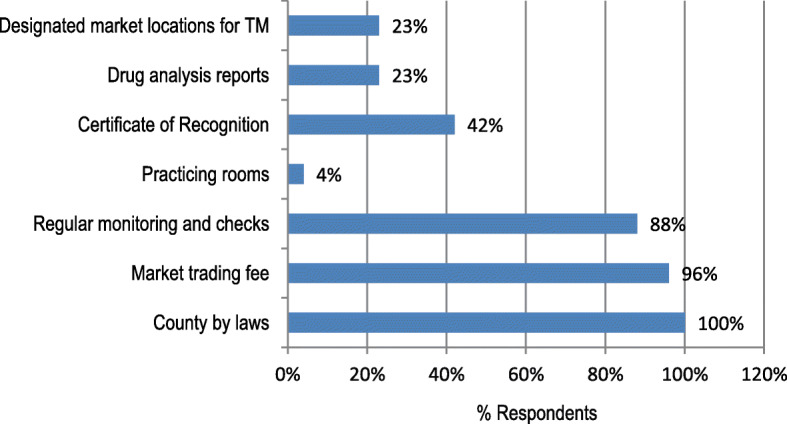
Modern governance practices observed by TM stakeholders in the sampled medicine markets

The modern governance practices were not significantly different in all the market centres surveyed, *p* (0.080) > 0.05 in the surveyed locations/markets (Table 6 and Fig. 3).

**Table 6:** The modern governance practices of traditional medicine in the selected TM markets. The number of interviewed respondents from each traditional medicine market is indicated in brackets

Modern governance practices (MGPs)	Nairobi (13)	Eldoret (3)	Kakamega (5)	Makutano (3)	Kitale (5)	Luanda (6)	Moi's Bridge (1)	Yala (1)	Arror (2)	Totals
Designated market locations	0	0	1	0	0	6	0	0	0	7
Drug analysis reports	13	0	3	0	1	3	1	0	0	21
Certificate of recognition	13	0	3	0	1	3	1	0	0	21
Practising rooms/spaces	0	0	2	0	0	6	0	0	0	8
Regular monitoring and checks	13	3	5	3	5	6	1	1	2	39
Market trading fee	13	3	5	3	5	6	1	1	0	37
County by laws	13	3	5	3	5	6	1	1	0	37
Total	65	9	24	9	17	36	5	3	2	170

**Fig. 4:**
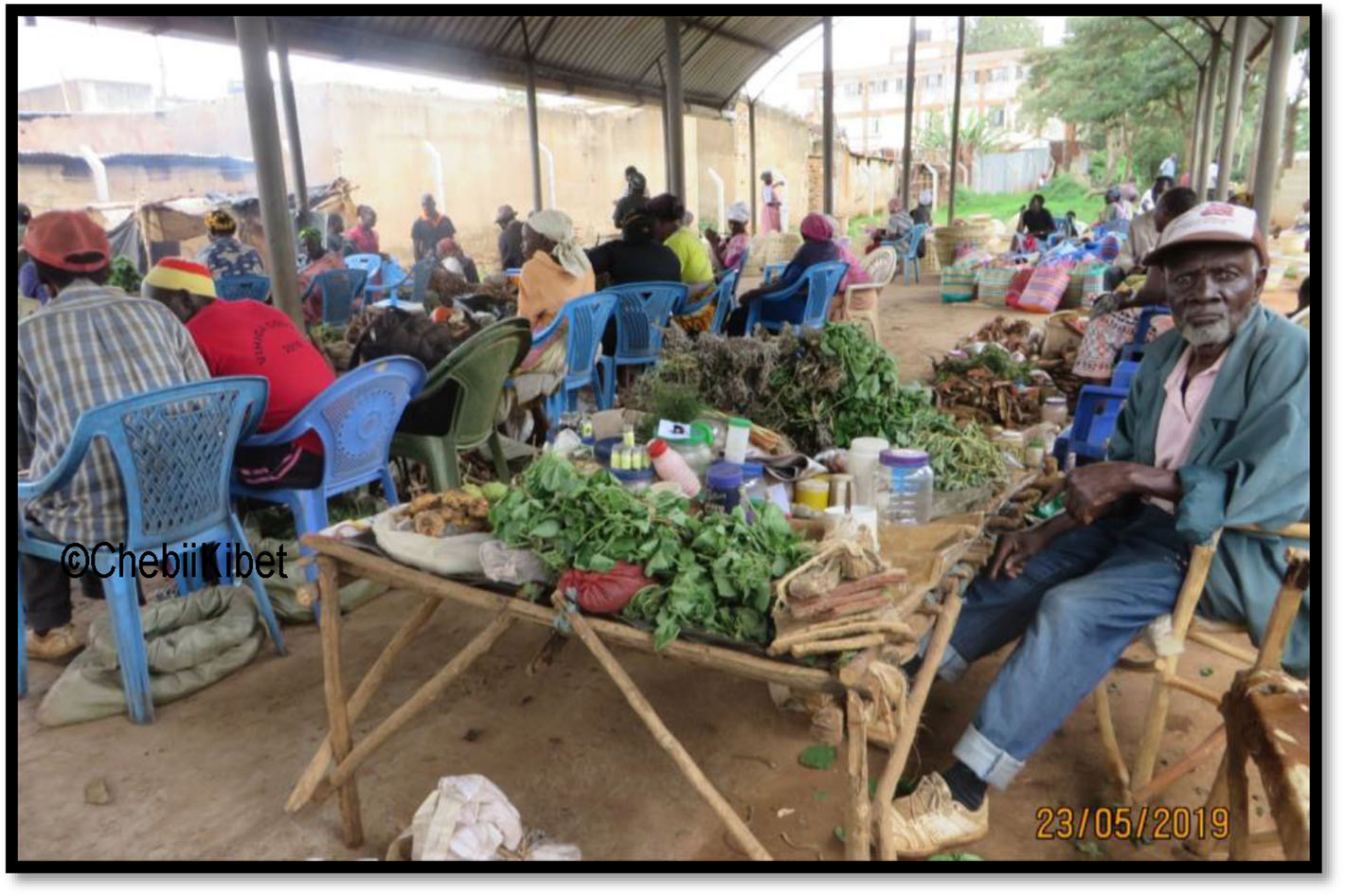
A sheltered traditional medicine market in Luanda, Vihiga County, Kenya

Descriptively, all interviewed respondents perceived that county-by-laws were fully observed by all practising traditional medicine practitioners in order to freely trade or practice. In addition, the TMPs also observed total compliance (96%) in the payment of market trading fee in order to avoid unnecessary punishment or penalties from the county government authorities. Majority of the respondents (88%) were in favour of regular monitoring and checks in the area of traditional medicine and quality control. However, lack of designated medicine market spaces (23%) and practising rooms (4%) were notable bottlenecks in the TM practice. Finally, most of the traded medicinal plants have not been analysed (Fig. 4).

### Traditional governance practices

**Fig. 5:**
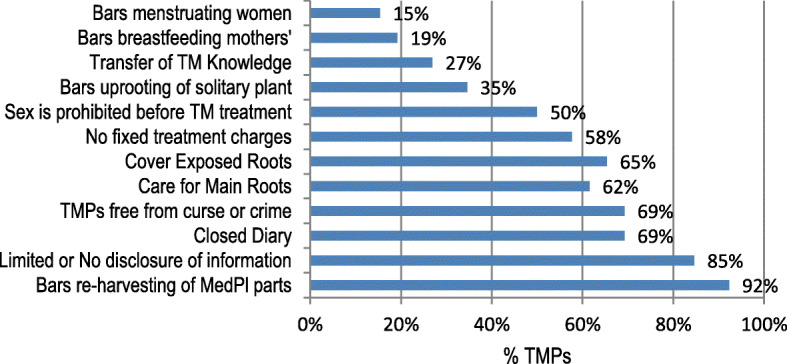
The traditional governance practices that regulate traditional medicine trade and practice

The traditional governance practices were not significantly different in all the market centres surveyed, *p* (1.000) > 0.05. The traditional governance practices emanated from diverse ethnic cultures of the traditional medicine practitioners and largely contribute to the informal trade practices. Descriptively, majority of the traditional medicinal practitioners (Fig. 5) are against re-harvesting of freshly harvested medicinal plants (92%) and backed limited or no disclosure of traditional medicine knowledge (85%). The conservative nature of traditional medicine practice bars breastfeeding mothers (19%) and menstruating women (15%) from practising TM. Some traditional medicine practitioners were guided by environmentally conscious decisions, for instance, not supporting uprooting of solitary medicinal plants (35%), caring for main plant roots (62%) and covering of exposed roots with mounds of soil (65%). These traditional governance practices also touched on morality, purity and cultural beliefs, for instance, TMPs were not allowed to engage in sexual intercourse in the entire treatment period (50%) to ensure purity and hasten patient healing, absence of fixed treatment charges (58%) to allow patients pay what they can afford, only TMPs free from curse or crime (69%) were allowed to practice and finally a traditional medicine practitioner should not disclose his medicine collection diary or rather adopt a closed diary in gathering of traditional medicine (69%).

### Secrecy/limited or no disclosure of traditional medicine knowledge

**Fig. 6:**
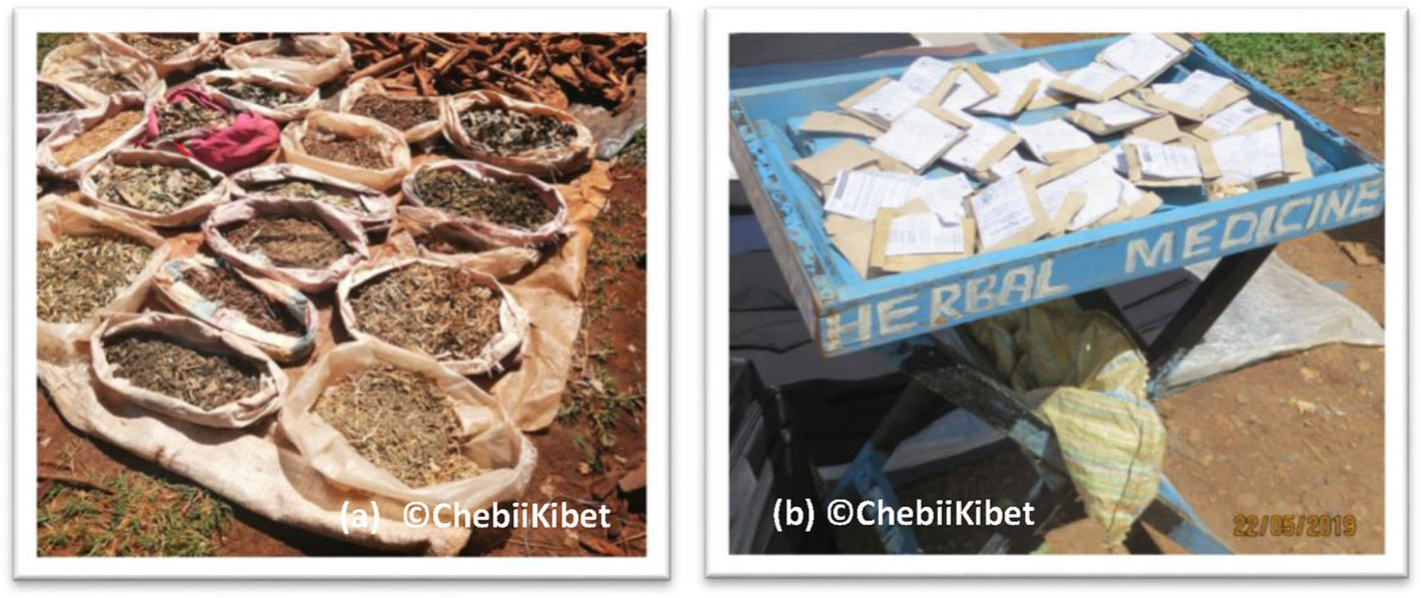
**a** Traditional medicine plants displayed on the roadsides of Kitale, Trans Nzoia County showing crushed leaves, roots and twigs. **b** Packets of herbal remedies used to treat various ailments, the label shows the name of the disease and a predetermined dose, the plant name is often omitted

Semi-structured interviews, field observations and photographic evidence revealed that few traditional medicine practitioners (15%) were willing to disclose important traditional medicine knowledge to customers, researchers, regulators and even policy makers. In addition, willing respondents could also not divulge vital information but general TM knowledge. The mostly disclosed information include the medicinal plant use, plant parts used, mode of drug preparation, disease treated or condition managed. Mostly hidden information by TMPs includes the traditional medicine’s name, name of the medicinal plant and where it is collected. Most of the medicinal plants displayed in open-air markets (Fig. 6a, b) lacked proper morphological and floral taxonomic characters and therefore making it difficult for customers’/users/patients’/buyers’ and researchers to easily identify the traditional medicine plants traded.

### Preferences and liking for traditional medicine

**Fig. 7:**
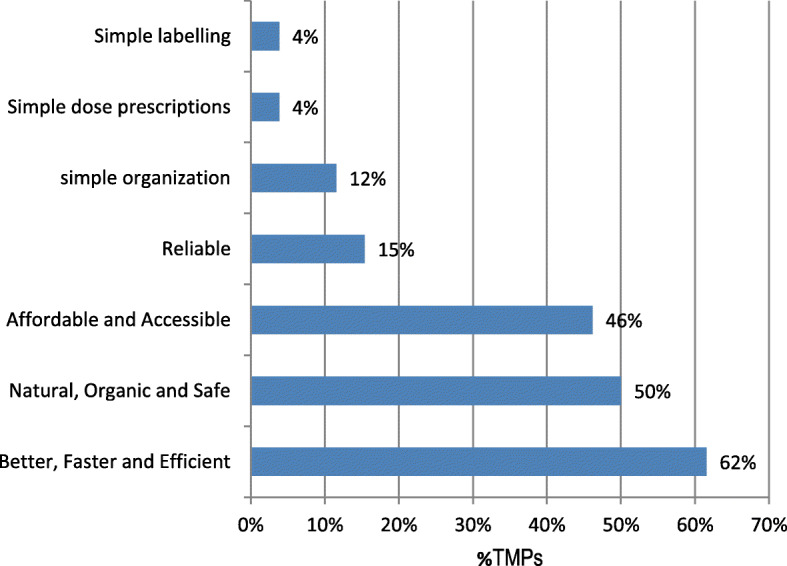
Perceptions of TMPs on customers/patients/clients preferences on the use of TM over conventional drugs

Based on experience and feedback from customers/patients/users of traditional medicine (Fig. 7), most traditional medicine practitioners believe that people prefer traditional medicine to conventional medicine because they are perceived to be better, faster in action and efficient (62%), natural, organic and safe (50%) and some think they are affordable and accessible (46%).

### Sources of traditional medicine knowledge

**Fig. 8:**
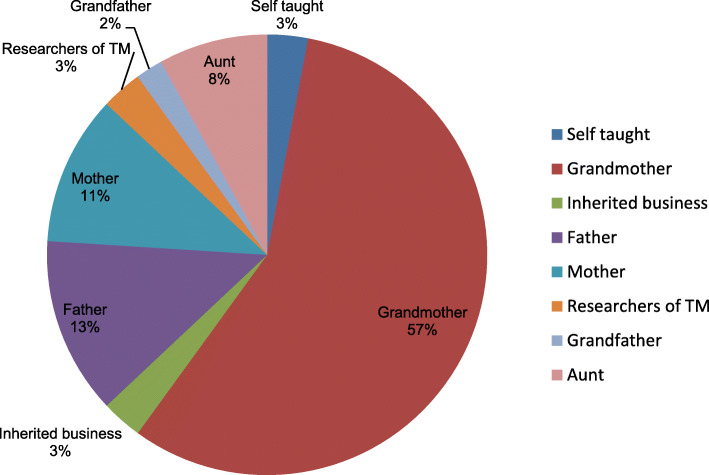
Sources of traditional medicine knowledge for most of the traditional medicine knowledge

**Fig. 9:**
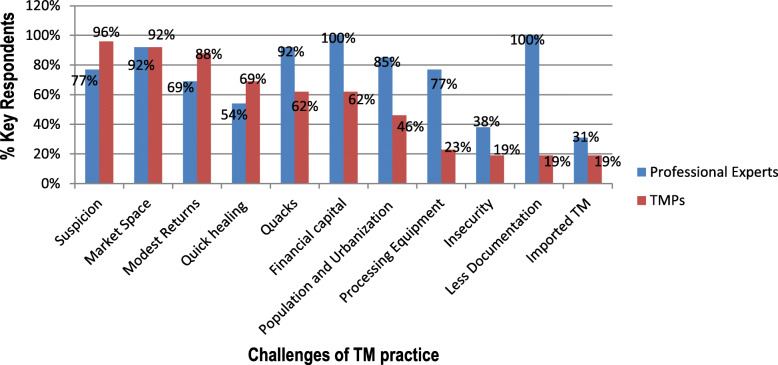
The main challenges afflicting traditional medicine sector in Kenya as perceived by both professional experts and traditional medicine practitioners

Most of the traditional medicine practitioners learnt their traditional medicine knowledge (Figs. 8 and 9) from their grandmothers (57%), some learnt from their fathers (13%), mothers (11%), aunts (8%) and few were self-trained (3%) and others learnt from field researchers (3%).

### Major challenges that affect traditional medicine in Kenya

**Fig. 10:**
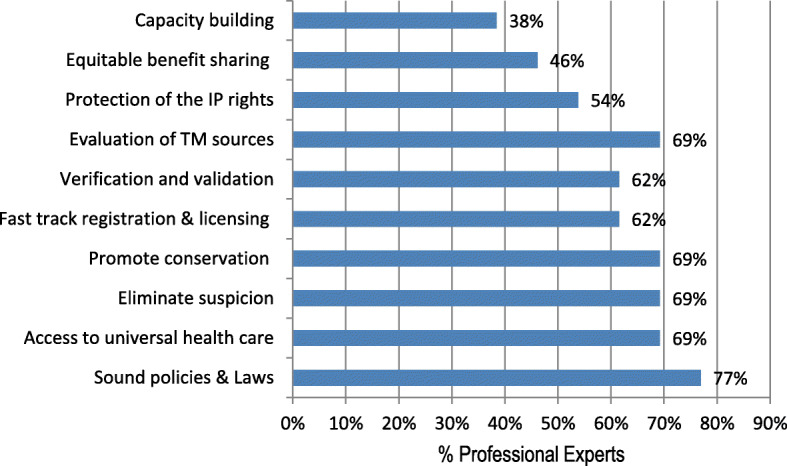
Some photographic evidence of formal and informal practices in the surveyed traditional medicine markets: **a** packaged TM products in small home-made envelopes in Kakamega town; **b** use of portable microphones and speakers in marketing of traditional medicine in Muliro Gardens, Kakamega town; **c** membership certificate of a TMP in Kitale town; **d** Certificate of Analysis of submitted products. Photographic images taken by Chebii Kibet.

The professional experts and traditional medicine practitioners expressed divergent opinions on what hails the traditional medicine industry (Fig. 10). Most traditional medicine practitioners perceived that lack of designated market spaces (92%) and suspicion between stakeholders as a result of mistrust (96%) were the main challenges. On the other hand, the professional experts perceived that lack of adequate documentation in traditional medicine (100%), inadequate financial capital (100%) and existence of incompetent TMPs (92%) were leading formal challenges hailing the TM industry.

### Formalization of the traditional medicinal practice

**Fig. 11:**
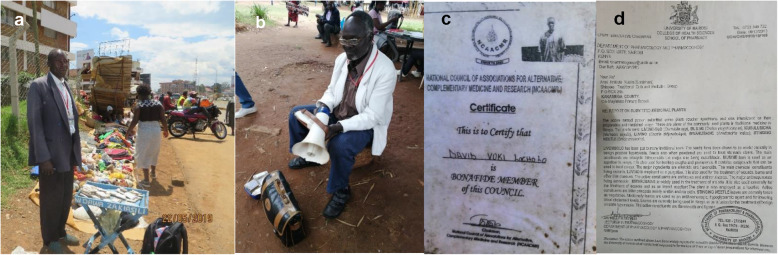
The perceptions of the sampled professional experts on the need for formalizing the traditional medicine sector

Majority of the professional experts (Fig. 11) perceived that formalization of traditional medicine practice is urgently needed in constituting important laws, by-laws and sound policies (77%). Although most of the raised thematic areas collated from the gathered perceptions had somewhat similar weight based on the need for formalization, most of the professional experts also perceived that formalization would bolster the promotion of traditional medicine and access to universal health care (69%), elimination of suspicion between stakeholders (69%), traditional medicine conservation (69%) and evaluation of traditional medicine sources (69%). Furthermore, these formal experts hold the view that formalization would hasten registration and licensing of the Traditional Medicine Practitioners (62%) and protection of the IP rights (54%).

### Formal and informal practices in the sampled traditional medicine markets

From field observation and study findings, there are far reaching legal and economic implications of the traditional medicine formal and informal market practices. Most of the traders in the surveyed traditional medicine markets do not have formal certification and licensing to operate but fully comply with county by-laws. All TMPs ensure they pay the mandatory market fees (KES, 30 per day) to avoid punishment from county authorities. From the regulators view point, possession of a Certificate of Recognition or Registration is an indication of competence in traditional medicine and not a license to practice. The surveyed markets of Western Kenya therefore represent a cocktail of both formal and informal practices where each practice plays a key role in the traditional medicine market economy (Table 7). The formal practices in the markets involve traditional medicine traders working within the confines of national laws and county by-laws, and enjoy recognition from the Department of Culture. All traditional medicine practitioners are also informally compliant to the traditional governance practices. In addition, almost all market activities are formally authorized by local administration authorities despite the informal nature of market transactions. The market spaces do not provide sufficient space for proper drug administration, patient handling and monitoring but are platforms for offering oral medicine prescriptions. This exposes patients to risks associated with distortions in drug dosage prescriptions in the markets and the actual drug doses taken at home.

**Table 7:** The observed formal and informal practices in the sampled traditional medicine markets of Western Kenya

Formal practices	Market centre	Informal practices	Market centre
Partial labelling (on paper packets or envelopes containing powdered TM product) and simple dosage prescriptions	Kakamega, Kitale	Oral or verbal dosage prescriptions	All TM markets
Packaged medicine on plastic bottles, paper envelopes or polythene bags	Eldoret, Kitale, Kakamega, Luanda and Moi’s Bridge	Simple display of traditional medicine on polythene bags, used fertilizer bags, baskets and mats on floor surfaces, make-shift tables or stands	All TM markets
Certification report on analysed medicinal plant samples	Kakamega, Kitale, Moi’s Bridge	Use of local languages and vernacular names of traditional medicine	All TM markets
County officers issuance of receipts on market fees paid by TM traders	All TM markets	Sale of fresh and dried plant materials in chopped, ground form or whole plant material, twigs, leaves, roots, flowers, tuber; and animal products. Sourcing of plant materials from the wild	All TM markets
Possession of Certification of Recognition/Practice from the Department of Culture	Kakamega, Kitale and Moi’s Bridge	No receipts issued on TM sales	All TM markets
Use of portable microphones/speakers and recorded audio to advertise medicinal products to pedestrians and commuters	Kakamega	No information on probable side effects	All TM markets
Market issues, concerns and TMPs welfare channelled through Traditional Herbalists and Practitioners Associations; Modest membership fee is charged	Luanda, Kitale	No scientific evidence on drug combinations and potential synergies	All TM markets
Sourcing of supplies from field collectors and village based herbalists	Makutano, Kakamega, Yala and Eldoret	Most traditional medicine practitioners’ associations are headed by male practitioners	All TM markets
Products diversification including trade on non-medicinal products: beads, calabashes, sweets/candy, snuff tobacco and processed imported herbal products	Eldoret, Kakamega and Kitale	Medical decisions are based on experience and apprenticeship	All TM markets
A money-driven cultural-based enterprise	All TM markets	Traditional medicine knowledge passed orally	All TM markets
Environmental consciousness and protection	All TM markets	Critical TM questions addressed by older practitioners	All TM markets
TM trade governed by county by-laws, national laws and policies	All TM markets	TM practices governed by strict unwritten traditional laws, norms, customs, taboos and beliefs	All TM markets

## Discussion

Elderly women dominated the traditional medicine markets and this was attributed to the need for traditional medicine experience and adherence to stringent traditional governance practices. These undocumented practices practically lock out young inexperienced women practitioners from active traditional medicine practice or trade unless under supervised apprenticeship. The traditional governance practices bars young women practitioners who are still nursing babies, actively breastfeeds or still undergo menstruation (Fig. 5, Table 8). Few studies also reported women dominance in the traditional medicine industry [87, 88]. However, unique scenarios occur where men become dominant in the traditional medicine market surveys due to religious and cultural reasons. For example, a religion that bar women from publicly interacting with male strangers [10]. The mean age of sampled traditional medicine practitioners in the survey was 64 years with an average experience of 25 years. This provided an indication that the traditional medicine industry is dominated by experienced and knowledgeable practitioners who have accumulated enormous traditional medicine knowledge and have earned huge public trust.

**Table 8:** The traditional governance practices governing traditional medicine, TMPs, trade and practice. The number of interviewed respondents from each market location is indicated in brackets

Traditional governance practices (TGPs)	Eldoret (3)	Kakamega (5)	Makutano (3)	Kitale (5)	Luanda (6)	Moi's Bridge (1)	Yala (1)	Arror (2)	Totals
Bars menstruating women	3	1	3	0	3	0	1	2	13
Bars breastfeeding mothers	3	1	3	0	3	0	0	2	12
Transfer of TM knowledge	3	5	3	5	4	0	1	2	23
Bars uprooting of solitary Medicinal plants	3	3	3	4	3	1	1	2	20
Sex is prohibited before treatment	3	2	3	4	2	0	0	2	16
No fixed treatment charges	3	2	3	0	1	1	1	2	13
Cover exposed roots	3	4	3	2	4	1	1	2	20
Care for main roots	3	4	3	2	4	1	1	2	20
TMPs should be free from crime or curse	3	2	3	0	3	0	1	2	14
Closed diary	2	2	3	1	3	1	1	2	15
No or limited treatment charges	3	2	2	1	5	1	1	2	17
Bars re-harvest of same medicinal plant	1	1	2	0	3	1	1	2	11
Total	33	29	34	19	38	7	10	24	194

Pearson's chi-square (77) = 34.3683, *p* = 1.000

Sex, gender and tribal-cultural inclinations had a bearing on the overall representation in some market centres. For instance, Makutano (West Pokot County), Eldoret (Uasin Gishu County) and Yala (Siaya County) market centres were dominated by women traditional medicine practitioners. The Kitale market in Trans Nzoia County was dominated by male traditional medicine practitioners interviewed (Table 4).

Most traditional medicine practitioners learnt their traditional medicine knowledge from their grandmothers (57%) than from the rest of the family members. The traditional medicine trade and practice was also characterized by suspicion and secrecy which was evident in all traditional medicine markets. Most traditional medicine traders were not willing to share vital traditional medicine knowledge. The relationship between professional experts and traditional medicine practitioners continue to be hampered by suspicion and secrecy. Local TMPs lack of willingness to disclose vital TM information is fuelled by incessant fear of losing hard-earned TM knowledge passed over generations. Secrecy and suspicion in the TM trade and practice also contribute to the resistance towards complete formalization of the TM industry.

**Fig. 12:**
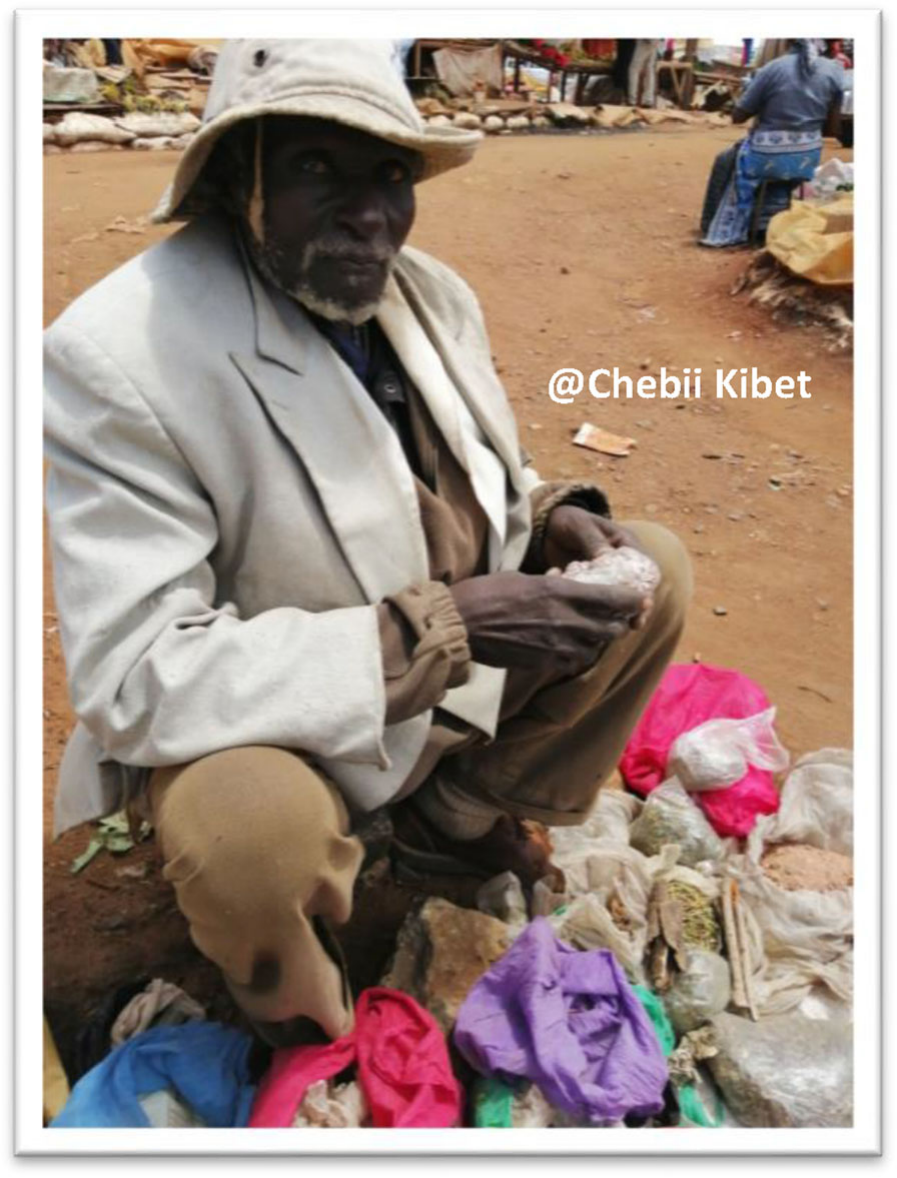
Mzee Job Budol Ole Ndinya, selling his traditional medicine on the streets of Moi’s Bridge market

The modern governance practices in the surveyed traditional medicine markets of Western Kenya were found not to be significantly different. It was observed that proper recognition of traditional medicine through clear legal and sound policies is not enough. Of equal importance is an effective implementation of set laws and efficient monitoring systems to ensure quality, purity and safety. A series of awareness campaigns and programmes are needed to educate registered TMPs on significant laws and adopted policies [89]. Standardized regulatory policies on herbal medicines are essential for global acceptance [90]. It is perceived that the existing laws and policies did not receive significant contribution from the traditional medicine ractitioners. Good laws and policies are key in the regulation of both domestic and international trade in traditional medicine, conservation and protection of medicinal plants and sustainable resource management [91]. The presence of unregistered TMPs (58%) heightens the need for stringent monitoring, tight checks and proper vetting of the traditional medicine practitioners or traders to rid the market of incompetent traditional medicine practitioners popularly dubbed ‘quacks’ (Fig. 12).

In most parts of the African continent, the traditional medicine practice is associated with witchcraft. These perceptions affect the development and progress of the traditional medicine industry. The colonial Witchcraft Act of 1925, cap 67 Laws of Kenya created more harm than good in the advancement of traditional medicine. Most traditional medicine practitioners were scared to openly practice and feared to be associated with witchcraft since it came with heavy fines and punishment.

The traditional governance practices were not significantly different in the surveyed traditional medicine markets despite the varying cultures of the people in the study areas. Traditional governance practices were more pronounced in Arror, Makutano, Yala and Eldoret medicine markets and less pronounced in Kakamega and Kitale medicine markets. Traditional medicine is not only utilized by the rural population but by users in the urban and peri-urban areas where access to modern medicines is not a challenge. Thus underscoring the need to integrate traditional medicine into the primary health care [15]. Lack of designated market spaces or practising rooms for traditional medicine practitioners forced most of the practitioners to ply their trade on roadsides or on urban streets. Informal markets are exposed to contamination by dusts, dampness and several dirt conditions that may decrease the quality and purity of traditional medicine. Local medicine research and relevant teaching institutions should work closely with traditional medicine practitioners in determining traditional medicine efficacy, active drug constituents, dosage determination and enlist probable side effects of commonly traded traditional medicine.

The quest for the formalization of TM trade and practice needs a non-elitist and multi-sectoral approach that is all-inclusive and values input from all major stakeholders. Good regulations governing harvesting, trade and practice should draw important elements from traditional customs, statutory bodies and relevant institutions [45]. A careful approach should be taken in the TM trade legislation to preclude over-regulation and avoid under-regulation so as not to destabilize the informal markets. The need for legitimization and rationalization of traditional medicine practices is perceived to be a key step towards complete formalization of the traditional medicine industry [92].

## Conclusion

Modern governance practices continue to receive a lot of attention and priority particularly from formal stakeholders and policy makers. Similar attention should be accorded to the traditional governance practices which also play a significant role in the governance of traditional medicine. Traditional governance practices constitute a system of informal and unwritten regulations usually derived from the socio-cultural beliefs of the local communities. They are invaluable in the regulation of virtually all TM informal practices and also serve as the initial vetting mechanism of competent traditional medicine practitioners and traders.

The availability of good laws and policies alone is never enough; there is a need for education and awareness campaign programmes, strict implementation processes and regular monitoring of the TM industry. Generally speaking, TMPs show unwavering compliance on both national and county-by-laws. However, enacted laws rarely cover critical aspects of traditional medicine, medicinal plants, processing of herbal products. The feeling of exclusion by the traditional medicine practitioners heightens suspicion, mistrust and slows advancement and integration of TM. Integration of traditional governance practices into the formal regulatory frameworks or harmonization of both modern and traditional regulations may bolster the traditional medicine industry.

For improved governance of traditional medicine, I recommend that

- Traditional medicine practitioners be included in the process of making laws and policies governing the traditional medicine
- Stringent regulatory procedures and monitoring should be observed to ensure the safety and efficacy of traditional medicine
- Integrate traditional medicine in the primary health care and encourage patient referrals to modern health care centres
- Practising/trading spaces and rooms should be set aside for traditional medicine practitioners to prevent contamination of the traditional medicine products
- Practising traditional medicine practitioners should be thoroughly vetted to rid the market centres of incompetent, unqualified and unethical practitioners popularly dubbed ‘quacks’
- Traditional governance practices should be integrated in the legal and regulatory frameworks of traditional medicine
- There is a need for laws and policies that govern traditional medicine markets and trade and allow for harmonization of both formal and informal practices of traditional medicine.

### Supplementary information

**Additional file 1.** Survey Questionnaire

## Data Availability

All the data are included in the manuscript.

## Appendix 1

**Sites/Websites and Documents Explored**

Convention on Biological Diversity https://www.cbd.int/doc/legal/cbd-en.pdf

Development Plan, Kenya (1989-1993).

Elgeyo Marakwet County https://elgeyomarakwet.go.ke/

Governing Traditional Medicine in Kenya https://www.tandfonline.com/doi/full/10.1080/00020184.2018.1452856

Health Bill 2015 http://kenyalaw.org/kl/fileadmin/pdfdownloads/bills/2015/HealthBill2015.pdf

Health Laws (Ammendment) Act, No. 5 of 2019 http://kenyalaw.org/kl/fileadmin/pdfdownloads/AmendmentActs/2019/HealthLaws__AmendmentActNo.5of2019.pdf

https://www.researchgate.net/publication/323267945_A_Critical_Overview_of_the_Health_Act_2017

Kenya National Drug Policy http://apps.who.int/medicinedocs/documents/s16443e/s16443e.pdf

KNBS 2019 https://www.knbs.or.ke/

Ministry of Gender, Sports, Culture and Social Services, Form DC1, 2003

Nagoya Protocol https://www.cbd.int/abs/doc/protocol/nagoya-protocol-en.pdf

National policy on traditional medicine and regulation of herbal medicines. Report of a WHO global survey, May 2005

Registration of Herbal and Complementary products. Guidelines to submission of applications. Pharmacy and Poisons Board, 2010. PPB copyright. https://www.pharmacyboardkenya.org/files/?file=herbal_guidelines.pdf

Sessional paper on Traditional Medicine in Kenya, 2009

Siaya County http://siaya.go.ke/

The Alma Ata Declaration https://www.who.int/publications/almaata_declaration_en.pdf

The Health Act No. 21 of 2017 http://kenyalaw.org/kl/fileadmin/pdfdownloads/Acts/HealthActNo.21of2017.pdf

The Health Bill, 2012 http://publications.universalhealth2030.org/uploads/health_bill_2012.pdf

The Health Laws (Ammendment) Bill, 2018, Kenya Gazette Supplement No. 36, National Assembly Bills, No. 14 http://kenyalaw.org/kl/fileadmin/pdfdownloads/bills/2018/HealthLaws_Amendment_Bill_2018.pdf

The Traditional and Alternative Health Practitioners Bill 2019

The Traditional Health Practitioners Bill (2014).

The Traditional Medicine and Medicinal Plants Bill (2010).

Trans Nzoia County https://www.transnzoia.go.ke/

Uasin Gishu County https://www.uasingishu.go.ke/

Vihiga County https://www.vihiga.go.ke/

West Pokot County http://www.westpokot.go.ke/

## Appendix 2

**GPS locations**

**Table:**

County	Sampled market	GPS coordinates	Altitude (m asl)
Uasin Gishu	Eldoret	N 00° 31.010′ E 035° 16.364′	2080
		N 00° 30.886′ E 035° 16.480′	2068
		N 00° 30.970′ E 035° 16.550′	2094
	Moi’s Bridge	N 00° 52.610′ E 035° 07.236′	1819
Elgeyo Marakwet	Arror	N 00° 57.838′ E 035° 36.967′	1049
Trans Nzoia	Kitale	N 01° 01.206′ E 035° 00.087′	1898
		N 01° 01.179′ E 035° 00.119′	1897
		N 01° 00.806′ E 035° 00.352′	1893
West Pokot	Makutano	N 01° 15.425′ E 035° 05.549′	2060
Kakamega	Kakamega	N 00° 17.231′ E 034° 45.206′	1556
		N 00° 17.055′ E 034° 45.219′	1565
		N 00° 17.231′ E 034° 45.342′	1572
Vihiga	Luanda	N 00° 01.427′ E 034° 35.167′	1505
Siaya	Yala	N 00° 05.301′ E 034° 32.387′	1389
