# Supplementary material for: The governance of traditional medicine and herbal remedies in the selected local markets of Western Kenya
Source: J Ethnobiol Ethnomed. 2020 Jun 26;16:39. doi: 10.1186/s13002-020-00389-x (PMC7320552; doi:10.1186/s13002-020-00389-x)
Supplement: Supplementary file 1 — Additional file 1. Survey Questionnaire [file 13002_2020_389_MOESM1_ESM.pdf]

QUESTIONNAIRE: THE GOVERNANCE OF TRADITIONAL MEDICINE AND HERBAL REMEDIES IN THE SELECTED LOCAL MARKETS IN KENYA.

Date of Interview: ……………………………….

Socio economic characteristics of the TMPs

Name ……………………………………………………………….

Sex of respondent Male [ ] Female [ ]

Age (years) ………………………………………

County of residence ……………………………………………………….

Phone number ……………………………...............

1. How long have you practiced traditional medicine? ………………………………
2. Who taught you traditional medicine?
3. What age category frequently visits for treatment?
4. How much are you charged by the county to trade/practice in the market centre?
5. Are you aware of the existing legal and policy frameworks?
6. Are you recognized or certified by the Department of Culture?
7. How much do you earn in a month from the traditional medicine practice?
8. Do you have any other source of income/livelihood?

   No [ ]

   Yes [ ]

   If YES, Mention these other sources of income

9. What challenges/concerns/threats do you face in your daily practice as a Traditional Medicine Practitioner?

   ----
10. What traditional governance practices governs the traditional medicine practice?
11. What modern governance practices regulate traditional medicine practice?
12. Are you a member of an active traditional medicine and herbal associations?

    No [ ]

    Yes [ ]

    If YES , what are their benefits?

13. Is there a conflict between traditional systems of governance and modern systems of governance? Can the two systems be harmonized for better governance and management of traditional medicine and trade in medicinal plants
14. Do you think the formalization of the TMP will improve the sector?

    YES [ ]

    NO [ ]

    State a reason ……………………………
15. What key areas are prioritized for training traditional medicine practitioners?
